# Understanding the psychosocial impact of colorectal cancer on young‐onset patients: A scoping review

**DOI:** 10.1002/cam4.4575

**Published:** 2022-02-11

**Authors:** Athena Ming‐Gui Khoo, Jerrald Lau, Xin‐Sheng Loh, Celeste Wen‐Ting Ng, Konstadina Griva, Ker‐Kan Tan

**Affiliations:** ^1^ Yong Loo Lin School of Medicine National University of Singapore Singapore Singapore; ^2^ Saw Swee Hock School of Public Health National University of Singapore Singapore Singapore; ^3^ Lee Kong Chian School of Medicine Nanyang Technological University Singapore Singapore

**Keywords:** behavioural science, colorectal cancer, psychosocial studies, QOL, quality of life

## Abstract

**Objective:**

The incidence of young‐onset (<50 years) colorectal cancer (CRC) has been increasing internationally. The psychosocial experience of younger cancer patients is vastly different from older patients, especially in domains such as financial toxicity, body image, and sexual dysfunction. What is unknown is the cancer type‐specific experience. The aim of the current scoping review was to examine (1) the psychosocial factors and/or outcomes associated with young‐onset CRC and (2) other determinants that influences these outcomes.

**Methods:**

A systematic search was conducted on four databases (PubMed, CINAHL, Scopus and PsycINFO) from inception to December 2020 using key terms and combinations. Primary literature that examined the psychosocial (e.g., quality‐of‐life, emotional, social, sexual) impact of young‐onset CRC were included.

**Results:**

A total of 1389 records were assessed by four reviewers, with a total of seven studies meeting inclusion criteria (*n* = 5 quantitative, *n* = 1 qualitative and *n* = 1 case series). All studies indicated there was significant psychosocial impact in younger CRC patients, including emotional impact, social impact, physical burden, sexual impact, work impact, unmet needs, financial impact and global quality of life. Three studies explored other determinants that influenced the psychosocial experience and found that socioeconomic background (e.g., being female, lower education), CRC treatment (e.g., chemotherapy) and health status were associated with worse psychosocial impact.

**Conclusions:**

Young‐onset CRC patients face severe psychosocial impact unique to this age group, such as self‐image and sexual impact. Social support services and resources needs to be uniquely tailored. More empirical investigations are required to understand its long‐term impact and influence of other psychosocial domains.

## Abstract 2

This scoping review of psychosocial impact in young‐onset colorectal cancer patients uncovered significant psychosocial impact which are in‐line with previous studies on young cancer (e.g., emotional impact, social impact, physical burden) as well as impact unique to colorectal cancer (e.g., self‐image, embarrassment with bowel movements and the impact of stoma). More empirical investigations are required to understand its long‐term impact and influence of other psychosocial domains.

## 1 BACKGROUND

Colorectal cancer (CRC) is one of the top cancers globally. While the majority of CRC cases are usually diagnosed in individuals above the age of 50 years, recent studies from the international body of literature have highlighted an increasing trend in the incidence of CRC in younger adults (young‐onset CRC; i.e., <50 years). This is most noticeable in the United States, but these trends are not restricted to the West. Developed Asian countries such as Taiwan, Korea, and Japan have all also experienced increases in incidence of young‐onset CRC within the past two decades.^1^, ^2^, ^3^, ^4^ Data from the GLOBALCAN 2020 database estimated that young‐onset CRC incidence has grown by 1%–4% per annum in developed nations.^5^ Moreover, according to the Global Burden of Disease Study 2019,^6^ colorectal cancer is in the top five main contributors to cancer‐associated death in adolescents and young adults.

It is intuitive that individuals with young‐onset CRC may undergo a vastly different psychosocial experience compared to older patients. International literature has shown that younger cancer survivors generally suffer from poorer quality of life (QOL), disruption to social and sexual health, and increased mental health‐related risks such as depression and anxiety.^7^, ^8^ A systematic review of young cancer patients in general has also identified four areas of concerns, namely physical well‐being, psychological well‐being, social well‐being and survivorship care, and that these outcomes are experienced differently from older adult counterparts, highlighting the need for age‐tailored care approaches.^9^ Younger survivors are also more likely to experience financial toxicity, a term defined as the psychological distress that results from the direct (e.g., medical costs) and indirect (e.g., loss of income) financial costs of cancer diagnosis and treatment.^10^, ^11^

While there is growing literature on the psychosocial impact of young‐onset cancer, prior work focused on cancer diagnosis in general and did not delve adequately into the unique challenges of specific types of cancer. For CRC in particular, patients may experience sexual dysfunction, urinary problems and loss of normal bowel function after undergoing rectal resection.^12^ Some patients may also require an ostomy, a surgically created opening to allow discharge of waste from body, which can negatively impact body image.^13^ Given that the incidence of young‐onset CRC continues to rise worldwide, there is a need to better understand the psychosocial impact of this specific disease in younger adults.

The present review aimed to summarise the existing body of literature and examine the key patient reported psychosocial factors and outcomes that are associated with CRC in young‐onset patients. We sought to answer the following research questions: (1) What are the patient‐reported psychosocial factors and/or outcomes associated with CRC in young‐onset patients, and (2) what other determinants influence these psychosocial factors and/or outcomes in young‐onset patients?

## 2 METHODS

**FIGURE 1:**
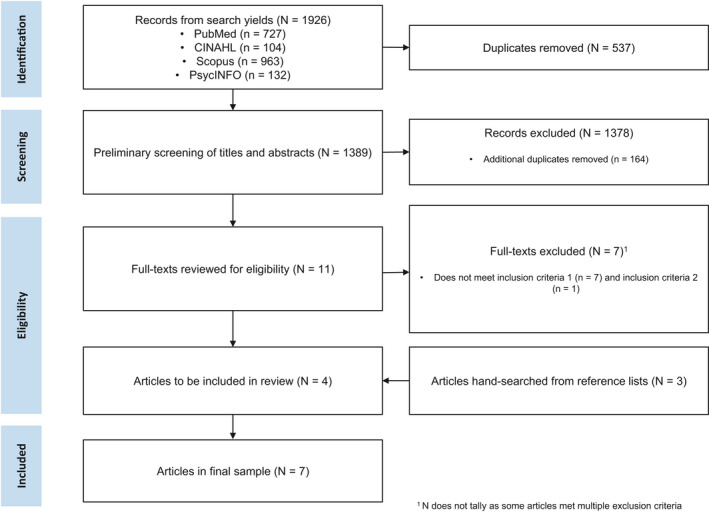
PRISMA flow chart illustrating search strategy used to identify eligible studies for inclusion

This scoping review was conducted in accordance with the Preferred Reporting Items for Systematic Reviews and Meta‐Analyses (PRISMA) and with a flow diagram (see Figure 1) and was registered on PROSPERO (ID: CRD42021235261) prior to conducting the systematic search.

### 2.1 Search strategy

A systematic search of four databases (PubMed, CINAHL, PsycINFOand Scopus) was conducted from database inception to December 2020. Given that not all articles examining psychosocial‐related factors might have used keywords that fit into the Medical Subject Headings (MeSH) database for terms relevant to 'psychosocial' due to the broad definition of this domain, we opted for a more general search strategy. The search strategy therefore only contained terms relevant to 'colorectal cancer' and 'young onset', allowing us to maximise search yields and screen through all articles relevant to young‐onset CRC. An example of the search strategy (for PubMed) is listed below:

('Colorectal neoplasms'[MeSH]) AND ('early onset' OR 'young onset')

For databases without access to MeSH search operators (CINAHL, PsycINFO, and Scopus), we manually entered all entry terms within the 'colorectal neoplasms' MeSH as individual search terms, as stated below:

('Colorectal Neoplasm' OR 'Neoplasm, Colorectal' OR 'Neoplasms Colorectal' OR 'Colorectal Tumors' OR 'Colorectal Tumor' OR 'Tumor, Colorectal' OR 'Tumors, Colorectal' OR 'Colorectal Cancer' OR 'Cancer, Colorectal' OR 'Cancers, Colorectal' OR 'Colorectal Cancers' OR 'Colorectal Carcinoma' OR 'Carcinoma, Colorectal' OR 'Carcinomas, Colorectal' OR 'Colorectal Carcinomas') AND ('early onset' OR 'young onset')

### 2.2 Study inclusion and exclusion criteria

All quantitative and qualitative studies were included if (1) the study sample included young‐onset CRC patients (i.e., below the age of 50 years) or included a stratification of a younger onset sample in comparison with an older sample of CRC patients, and (2) the study examined patient reported psychosocial factors and/or outcomes associated with colorectal cancer diagnosis and/or treatment in young‐onset patients. While young‐onset CRC was defined as patients below the age of 50, we decided to also include studies in which analyses and findings between the young‐onset and older sample of CRC patients were clearly stratified as this permitted young‐old comparisons.

Studies were excluded if (1) they were grey literature (i.e., not peer‐reviewed), (2) the full text of the study was not published in English, or (3) did not contain analysis of primary data (e.g., study was a review, meta‐analysis, editorial, or commentary).

For the current study, we defined psychosocial using the standing definition provided by the National Cancer Institute (NCI) which states that the psychosocial experience encapsulates 'the mental, emotional, social, and spiritual effects of a disease'.^14^

### 2.3 Study selection, data extraction and analysis

The search strategy was applied to each of the four databases by one co‐author (AMK). All retrieved citations were downloaded into EndNote X8 where duplicates were removed. Four co‐authors (AMK, JL, XL and CWN) conducted an independent preliminary screening of titles and abstracts using the inclusion/exclusion criteria. During this process, additional duplicates that were not picked up by the EndNote X8 software were also found and manually removed. Studies that met the inclusion criteria underwent a full text screening by two co‐authors (AMK and JL). Any cases of disagreement regarding study inclusion were resolved via discussion with consensus by all five authors.

Four co‐authors (AMK, JL, XL and CWN) performed the data extraction for the final sample of included studies. Information such as study design and methodology, country of origin for the study, sample characteristics and key findings were extracted. Key findings extracted included results relevant to the research questions of this review, such the psychosocial experience of younger CRC patients, instruments used to measure psychosocial factors, other factors associated with the psychosocial outcomes measured, as well as any other relevant key findings of interest. Findings were narratively summarised from each included article into a table. Through discussion and mutual agreements, two co‐authors (AMK and JL) inductively grouped similar psychosocial factors found across the findings into common narrative categories. The PRISMA flow diagram depicting the study selection process can be found in Figure 1.

### 2.4 Statistical analysis

No statistical analysis was conducted as this is a scoping review.

### 2.5 Risk of bias

Quality appraisal of the studies included in the final sample was performed using the relevant Joanna Briggs Institute (JBI) critical appraisal tools by two co‐authors (JL and AMK) independently. This tool evaluates methodological quality of studies by examining the possibility of bias in design, conduct and analysis.^15^

## 3 RESULTS

### 3.1 Quality appraisal of the included studies

Using the JBI critical appraisal checklists appropriate to the respective study designs, each of the seven included studies were appraised. Appraisal scores were calculated by the proportion of items that met the criteria of each respective checklist after excluding items that were not applicable. All seven included studies showed high appraisal scores ranging from 70% to 100%, suggesting a relatively low risk of bias (see Tables 1, 2, 3, 4 for detailed results of the quality appraisal).

**TABLE 1:** JBI critical appraisal checklist results (cross‐sectional studies)

Checklist questions	Bailey et al (2014)	Downing et al (2015)	Perl et al (2016)
1. Were the criteria for inclusion in the sample clearly defined?	Yes	Yes	Yes
2. Were the study subjects and the setting described in detail?	Yes	Yes	Yes
3. Was the exposure measured in a valid and reliable way?	Yes	Unclear	Yes
4. Were objective, standard criteria used for measurement of the condition?	Yes	Yes	Yes
5. Were confounding factors identified?	Yes	Yes	Yes
6. Were strategies to deal with confounding factors stated?	Yes	Yes	Yes
7. Were the outcomes measured in a valid and reliable way?	Yes	Yes	Yes
8. Was appropriate statistical analysis used?	Yes	Yes	Yes
Overall appraisal	Include	Include	Include

**TABLE 2:** JBI critical appraisal checklist results (qualitative studies)

Checklist questions	Blum‐Barnett et al (2019)
1. Is there congruity between the stated philosophical perspective and the research methodology?	Unclear
2. Is there congruity between the research methodology and the research question or objectives?	Yes
3. Is there congruity between the research methodology and the methods used to collect data?	Yes
4. Is there congruity between the research methodology and the representation and analysis of data?	Yes
5. Is there congruity between the research methodology and the interpretation of results?	Yes
6. Is there a statement locating the researcher culturally or theoretically?	No
7. Is the influence of the researcher on the research, and vice‐ versa, addressed?	No
8. Are participants, and their voices, adequately represented?	Yes
9. Is the research ethical according to current criteria or, for recent studies, and is there evidence of ethical approval by an appropriate body?	Yes
10. Do the conclusions drawn in the research report flow from the analysis, or interpretation, of the data?	Yes
Overall appraisal	Include

**TABLE 3:** JBI critical appraisal checklist results (case series)

Checklist questions	Caffo (2002)
1. Were there clear criteria for inclusion in the case series?	Yes
2. Was the condition measured in a standard, reliable way for all participants included in the case series?	Yes
3. Were valid methods used for identification of the condition for all participants included in the case series?	Yes
4. Did the case series have consecutive inclusion of participants?	Yes
5. Did the case series have complete inclusion of participants?	Unclear
6. Was there clear reporting of the demographics of the participants in the study?	Yes
7. Was there clear reporting of clinical information of the participants?	Yes
8. Were the outcomes or follow up results of cases clearly reported?	Yes
9. Was there clear reporting of the presenting site(s)/clinic(s) demographic information?	Yes
10. Was statistical analysis appropriate?	Yes
Overall appraisal	Include

**TABLE 4:** JBI critical appraisal checklist results (cohort studies)

Checklist questions	Mack et al (2016)	Sanford et al (2015)
1. Were the two groups similar and recruited from the same population?	Yes	Yes
2. Were the two groups similar and recruited from the same population?	Yes	Yes
3. Was the exposure measured in a valid and reliable way?	Yes	Yes
4. Were confounding factors identified?	Yes	Yes
5. Were strategies to deal with confounding factors stated?	Yes	Yes
6. Were the groups/participants free of the outcome at the start of the study (or at the moment of exposure)?	Unclear	Yes
7. Were the outcomes measured in a valid and reliable way?	Yes	Yes
8. Was the follow up time reported and sufficient to be long enough for outcomes to occur?	NA	Unclear
9. Was follow up complete, and if not, were the reasons to loss to follow up described and explored?	NA	NA
10. Were strategies to address incomplete follow up utilised?	NA	NA
11. Was appropriate statistical analysis used?	Yes	Yes
Overall appraisal	Include	Include

### 3.2 Descriptive characteristics of included studies

The seven included studies comprised 23,907 CRC patients, of whom there were a total of 2552 young‐onset CRC patients recruited in four countries. Among these studies, one (*N* = 1; 14.3%) was a qualitative investigation using semi‐structured interviews^16^; another was a case series^17^ (*N* = 1; 14.3%). The remaining five studies examined psychosocial impact quantitatively: three utilised cross‐sectional designs,^18^, ^19^, ^20^ and the other two used prospective cohort designs.^21^, ^22^ The majority (*N* = 6, 85.7%) of the studies included were conducted in countries with predominantly western settings, with one study^20^ being conducted in Israel. All but one of the studies^17^ were published within the last decade. Five studies^17^, ^18^, ^19^, ^21^, ^22^ (71.4%) included both late‐onset and young‐onset colorectal cancer patients while the remaining two studies^16^, ^20^ only comprised of the latter. With regards to the age criteria for young‐onset, two studies^20^, ^21^, ^22^ (28.6%) defined young‐onset patients as 40 years and below at diagnosis, three studies^16^, ^18^ (42.8%) defined it as below 50 years, and the remaining two studies^17^, ^19^ as below 55 and 65 years respectively. Half (*n* = 4, 57.1%) of the studies were conducted on patients who were at an average of less than 3 years since diagnosis, one study examined patients who had survived 5 years since diagnosis, while the remaining two studies^20^ (28.6%) did not specify the length of survivorship.

### 3.3 Psychosocial experience of young‐onset CRC

The psychosocial impact experienced by young‐onset CRC patients (Table 5) was broadly categorised into emotional impact, social impact, physical burden, sexual impact, work impact, unmet needs, financial impact and global quality of life.

**TABLE 5:** Descriptive summary of key findings from included studies

Study authors and country of publication	Purpose and design of study	Sample characteristics	Psychosocial scale(s) used	Key findings	Key terms
Bailey et al (2014) United States	To examine the relationship onset of colorectal cancer and long‐term function and symptoms Cross‐sectional	*N* = 830 (282 are Young‐onset survivors and 548 Late‐onset survivors) Age: Mean of young‐onset survivors = 43.4, mean of late‐onset survivors = 62.6 Age range was not specified. Gender: Female (467), Male (363) Type of cancer: Colorectal Cancer	European Organisation for Research and Treatment of Cancer CRC module (EORTC CR 29)	Younger‐onset survivors (age 18–50 years) reported worse (*p* < 0.05) anxiety, body image, and embarrassment with bowel movements in comparison to older‐onset patients (more than age 50 years). However, older patients experience greater sexual dysfunction.	Emotional distress Sexual impact
Blum‐Barnett et al (2019) United States	To examine the relationship between cancer diagnosis among early‐onset CRC survivors and financial or QoL impact Qualitative Analysis	*N* = 14 Age: Age range: <50 Mean age was not specified. Gender: Did not specify Type of cancer: Colorectal Cancer	NA	Early‐onset CRC survivors (less than age 50 years) experience 1) financial impact due to affected career trajectory, earning potential and performance, 2) QOL impact stemming from stress, strained relationship and lack of information, and 3) physical side‐effects	Financial impact Social impact Emotional distress Unmet needs
Caffo et al (2002) Italy	To assess toxicity and daily changes in QoL of CRC patients with postoperative pelvic radiotherapy (XRT) using diary card Case series	*N* = 27 Age: Median age of CRC patients = 63, Age range of CRC patients = 42–76 Gender: Female = 7, Male = 20 Type of Cancer: Rectal Cancer	European Organisation for Research and Treatment of Cancer Quality of Life Questionnaire‐30 Diary Card	Younger CRC patients (less than age 65 years) experience higher pain score (*p* < 0.001) during treatment in comparison to older CRC patients (age 65 years and more)	Physical burden
Downing et al (2015) England	To examine the health‐related quality of life (HRQL) of CRC patients diagnosed 12 to 36 months earlier and to identify factors associated with poor health outcomes upon CRC diagnosis Cross‐Sectional Study	*N* = 21,802 Age: CRC patients <55 years = 2040, CRC patients ≥55 = 19,762 Age range was not specified. Gender: Female = 9119, Male = 12,683 Type of cancer: Colorectal Cancer	EuroQol‐5D (5Q‐ED)	Young CRC patients (less than age 55 years) more likely to report more problem on more than one domains of HRQL (*p* < 0.001) in comparison to matched sample of general population.	Global Quality of life
Mack et al (2016) United States	To understand experiences with treatment decision‐making among young adults with cancer Prospective Cohort Study	*N* = 592 (148 are young patients, 44 are middle‐aged patients) Age: Mean age of young patients and middle age patients was not specified. Age range of young patients = 21–40 years, Age range of middle‐aged patients = 41–60 years Gender: Female = 351 (87 young patients, 264 middle‐aged patients), Male = 241 (61 young patients, 180 middle‐aged patients) Type of cancer: Colorectal Cancer = 476 (119 young patients, 357 middle‐aged patients), Lung Cancer = 114 (29 young patients, 87 middle‐aged patients)	Did not use validated scales for measurements	Young patients (age 21–40 years) experienced greater cancer treatment‐related worries (*p* = 0.02, in particular time spent away from family (*P* = 0.002) in comparison to older patients (age 41–60 years)	Emotional distress Social impact
Sanford et al (2015) Multi‐site	To examine symptom burden experienced by young CRC patients receiving treatment for CRC Cohort Study	*N* = 718 (681 are ≥40 years, 37 are ≤39 years) Age: Mean of young CRC patients = 35.1, mean of older CRC patients = 60.5 Age range was not specified. Gender: Female = 328, Male = 353 Type of cancer: Colorectal Cancer	5‐point Likert scale MD Anderson Symptom Inventory (MDASI)	Young CRC patients (age 39 years and less) were more likely to experience (*p* < 0.05) physical symptoms burden including moderate or severe pain, rash, nausea, fatigue, distress, drowsiness and shortness of breath in comparison to older CRC patients (age 40 years and above). They also experience greater symptom interference with general activity, mood, work, interpersonal relationships, and enjoyment in life.	Physical burden Emotional distress Social impact Work impact
Perl et al (2016) Israel	To characterise CRC patients' specific needs and quality of life concerns Cross‐Sectional Retrospective Survey	*N* = 50 Age: Mean age of CRC patients = 35.5 Age range = 20–49 Gender: Female,^26^ Male^24^ Type of cancer: Colon Cancer,^25^ Rectum Cancer,^15^ Gastric Cancer,^4^ Esophagus Cancer,^2^ Others^4^	12‐item Short‐form Health Survey (SF‐12, version 2) Cancer Rehabilitation Evaluation System (CARES) Sexual Functioning Summary Scale short form (SF) Cancer Survivors' Unmet Needs questionnaire (CaSUN)	Young CRC patients (less than age 40 years) experience worse QoL during treatment in comparison to their pre‐treatment scores (*p* < 0.005) in areas of physical symptoms such as diarrhoea, sexual dysfunction and sleeping disorders, as well as in psychosocial domains such as impaired function in occupational activities and coping with children. Scores improve post‐treatment in comparison to treatment scores but did not return to pre‐treatment levels. Common unmet needs reported by young CRC patients include nutritional counselling (70%), psychosocial support (44%) and supporting group (40%).	Sexual impact Physical burden Work impact Unmet needs Social impact

#### 3.3.1 Emotional distress

Emotional distress was one of the most common experiences, reported by four articles.^16^, ^18^, ^21^, ^22^ Findings in this category include any negative experience in mood and emotions as a result of CRC diagnosis or treatment. Three of the quantitative articles reported that in comparison to older samples, young CRC patients experienced significantly worse (*p* < 0.05) emotional distress. Specifically, one study^18^ utilised the European Organisation for Research and Treatment of Cancer CRC module (EORTC QLQ‐CR29) and identified that younger patients had poorer anxiety and body than older patients, with 57.1% (vs. 69.6% in older) and 73.9% of younger patients (vs. 81.8% in older) scoring high on functioning in the respective domains, and 46.5% of younger patients (vs. 27.8% in older) experienced embarrassment with bowel movements. Another study^22^ examined symptom interference with mood measured using the MD Anderson Symptom Inventory (MDASI) which found moderate or severe scores in 40.5% of younger patients in comparison to 15.3% of older patients. The last quantitative article^21^ found younger patients to report more treatment‐related worries but used an unvalidated scale. Finally, the qualitative study^16^ identified the domain of stress and revealed that one major source came from financial burdens.

#### 3.3.2 Social impact

Social impact was also reported by four studies.^16^, ^20^, ^21^, ^22^ Findings were grouped under this category if they entailed impaired functioning in maintaining social and familial relationships or performing social roles. Three articles were quantitative studies, with two of the articles reporting that younger CRC patients experienced significantly greater social impact in comparison to an older sample of CRC patients (*p* < 0.05). Specifically, one study^21^ used an unvalidated scale and reported that younger patients experience greater concerns with spending time away from family due to treatment while the other study^22^ used the MDASI and found that physical symptoms have greater interfere with interpersonal relationships in younger patients. The third quantitative study^20^ used the Cancer Rehabilitation Evaluation System (CARES) and found greater impact with regards to coping with children during treatment compared to their pre‐treatment functioning (*p* < 0.05). The remaining article was a qualitative study^16^ interviewing on young CRC patients and suggested that the social impact could be attributed to the cancer's physical side‐effects, the need for family members to provide care to the patient, and their inability to perform their social roles.

#### 3.3.3 Physical burden

Three studies^17^, ^20^, ^22^ examined the experience of physical burden by younger CRC patients, reporting on the physical symptoms and side effects due to disease and/or treatment. Two articles^17^, ^22^ revealed significantly greater (*p* < 0.05) experience of physical symptoms such as fatigue, nausea and pain in young CRC patients in comparison to an older sample, measured using the MDASI and a diary card validated by the authors in a pilot study. The third article^20^ used the CARES to measure symptom severity and found greater severity of symptoms (*p* < 0.05) such as diarrhoea, sleeping disorders and abdominal in young CRC patients during and post‐treatment in comparison to their pre‐treatment levels.

#### 3.3.4 Sexual impact

Only two studies^18^, ^20^ looked at the sexual impact of CRC on younger patients. Both studies examined sexual functioning, with one study^18^ using the EORTC QLQ‐CR29, which has been validated for use with CRC patients whereas the other study^20^ employed a generic instrument, namely the Sexual Functioning Summary Scale short form, which is validated with cancer patients but not specifically for CRC. The first found that older CRC patients actually experience greater sexual dysfunction (*p* < 0.05) in comparison to younger patients while the second reported that young CRC patients had significantly worse (*p* < 0.05) sexual dysfunction during treatment in relation to their pre‐treatment scores.

#### 3.3.5 Work impact

Two of the articles^20^, ^22^ reported findings that can be categorised under the domain of work impact (i.e., the impact of young CRC on one's ability to perform their role in occupational activities), both of which were quantitative by design. Both studies reported the decline in younger patients' ability to perform occupational activities, with the first article^20^ measuring the impact using CARES and finding greater difficulties during treatment in comparison to their pre‐treatment level, while the second article^22^ used MDASI and found greater difficulties in comparison to an older sample (*p* < 0.05).

#### 3.3.6 Unmet needs

Unmet needs were also identified in two articles.^16^, ^20^ Findings were grouped under this category if they are pertaining to any supports and/or resources that were lacking in young CRC patients. One article^20^ utilised the Cancer Survivors' Unmet Needs questionnaire (CaSUN) and found that 70.0% of young CRC patients reported nutritional counselling as an unmet need, followed by 44.0% and 40.0% reporting psychosocial support and supporting group as unmet needs, respectively. The other article was a qualitative study^16^ which highlighted that young CRC patients reportedly lack information regarding insurance, chemotherapy, ostomy and sexual side‐effects of their cancer and treatment.

#### 3.3.7 Financial impact

Financial impact was only reported by one qualitative study.^16^ Findings were included in this category if the concerns involve the impact of young CRC patients' diagnosis and treatment on the patient's finances. The study noted the experience of financial toxicity in young CRC patients, in that cancer diagnosis and treatment were seen as disruptive of their career prospects/trajectories and as damaging for their potential earnings.

#### 3.3.8 Global quality of life

Some of the above‐mentioned findings utilised QOL instruments but reported findings on the individual QOL domains such as emotional and social. This category consists of findings reporting general or global scores of young CRC patients on quantitative QOL measures. Only one study^19^ found that younger CRC patients are more likely to report more than one problem in QOL domains on the EuroQol‐5D scale in comparison to a matched sample from the general population (68.8% vs. 59.9%). The study did not report on the findings of each of the specific domains.

### 3.4 Factors influencing the psychosocial experience of young‐onset CRC

Three studies^19^, ^20^, ^21^ further explored factors that can influence the psychosocial experience of young‐onset CRC (Table 6). Socioeconomic background, CRC treatments and health status were the three factors found to significantly influence on the psychosocial experience of young CRC patients.

**TABLE 6:** Descriptive summary of factors influencing the psychosocial experiences of young CRC

Study authors	Factors influencing the psychosocial experiences of young CRC	Key terms
Mack et al (2016)	For both younger (age 21–40 years) and older‐onset (age 41–60 years) CRC patients, those with dependent children and lower educational attainment reported higher odds of having more treatment‐related worries (*p* = 0.02).	Socioeconomic background
Downing et al (2015)	While younger patients (less than age 55 years) reported greater problems with their health related QOL compared to older patients (age 55–84 years), for CRC patients in general, reporting of more than one problem had greater odds (*p* < 0.01) in patients with active or recurrent disease, three or more long‐term conditions, with stoma, living in deprived area, receiving chemotherapy and/or radiotherapy and being female.	Health status CRC treatments Socioeconomic background
Perl et al (2016)	In young CRC patients (less than age 40 years), female patients reported higher unmet needs for nutritional counselling and psychosocial support as compared to male patients (*p* < 0.05). Patients with multimodality treatment (surgery, chemotherapy, and radiation) also presented higher rates of unmet needs (*p* < 0.05).	Socioeconomic background CRC treatments

The most common factor was socioeconomic background, covered in all three articles. Using Fisher's exact test, one of the articles^20^ found that young female CRC patients were more likely to report more unmet needs in nutritional counselling and psychosocial support (*p* < 0.05). The findings for the remaining two articles included both young and older‐onset patients. One article^21^ reported that CRC patients with dependent children and lower educational attainment had a higher odds ratio of having more treatment‐related worries (*p* = 0.02), while the final article^19^ found greater odds of reporting problems in health related QOL for patients living in deprived areas and who were female (*p* < 0.01).

The next common factor was the type of CRC treatments. One article^20^ found that having chemotherapy and/or radiotherapy treatment in addition to surgery was associated with higher rates of unmet needs (*p* < 0.05) in young CRC patients. The other article^19^ found that chemo/radiotherapy was associated with greater problem on health related QOL for both young and older‐onset CRC patients, and additionally found that patients with stoma had greater odds of reporting problems with health related QOL (*p* < 0.01).

Finally, the influence of health status was examined in only one study.^19^ For both younger and older‐onset patients, poorer health related QOL was reported with greater odds (*p* < 0.01) in patients whose cancer was still active or in recurrence and had three or more chronic conditions.

## 4 DISCUSSION

To our knowledge, the current scoping review is the first to examine the psychosocial experience in young‐onset CRC patients specifically. While the incidence of young‐onset CRC cases has been increasing in recent years, literature understanding their unique psychosocial experience has remained sparse given that our review found only seven studies looking into this specific topic. Nevertheless, the articles included suggest a consensus that young‐onset CRC patients do experience poorer psychosocial outcomes in comparison to their baseline state, as well as to older‐onset CRC patients. In addition, our included articles suggest that these outcomes can be influenced by other socioeconomic and treatment‐related factors. However, there remain unexplored domains and gaps in the existing knowledge of the psychosocial impact of young‐onset CRC.

Young‐onset CRC diagnosis and treatment impacts multiple facets of the psychosocial experience of younger patients. Consistent with previous literature on the experience of young cancer patients in general,^9^, ^23^, ^24^ the articles included in this review also found adverse impact for young‐onset CRC patients in the emotional, social, and physical domains, general QOL as well as the experience of financial toxicity and unmet needs. Interestingly, one study found that sexual dysfunction is greater in older CRC patients but that finding can be attributed to age. Moreover, sexual dysfunction is a measure of objective capability and does not indicate subjective feelings and interpersonal impact towards this loss of sexual functioning. In general, these findings were consistent that young‐onset patients tended to fare worse when compared to older‐onset CRC patients, the young‐onset CRC patients' own baseline, and to the general population. While improvements were observed post‐treatment, it does not seem to return to baseline levels.

Our study also found certain experiences that are more significant to CRC than to other cancers. For example, the formation of an ostomy following colorectal surgery is sometimes necessary for CRC patients, which involves an external pouch containing the patient's stool.^25^, ^26^ Hence, it is understandable that under the domain of emotional impact, the young CRC experience is marked by reduced self‐image and embarrassment with bowel movements. Concerns regarding one's appearances tended to be greater for the younger population as they are a phase in life marked by greater levels of sexual and social activity.^27^ Finally, the experience of physical distress was also specific to cancer's region of origin, with bowel and urinary problems being commonly reported. Overall, while our findings were consistent with existing cancer literature, it also highlighted some experiences which may be unique to young‐onset CRC patients.

There are also certain unexplored domains reported in other cancers but not found in the included studies of this review. One such domain is the cognitive impact of CRC and its treatment, which has been reported for other cancers such as breast^28^ and a general sample of CRC patients.^29^ There is also a lack of utilisation of quantitative instruments dedicated to investigating psychosocial outcomes related to CRC. Only one study^18^ used the EORTC QLQ‐CR29, a measure of CRC‐related QOL which includes subscales such as body image and stoma‐related impact. Finally, some of the domains found in this review were only examined in one or two studies, such as sexual, financial, and work impact. Further research into these domains should be conducted to establish their significance.

While only one qualitative study^16^ was available, it highlighted the interrelatedness of the individual psychosocial domains. In the article, one of the sources of negative social impact experienced by young CRC patients was due to physical side‐effects and similarly, some of the reasons for poorer QOL were related to financial and social impact as well as lack of resources. Similarly, a recent meta‐synthesis on qualitative studies on young‐onset cancer also found that the domains of patients' experiences were bi‐directional and interrelated.^30^ The implication of this finding is that when understanding and tackling the psychosocial impact of CRC in young‐onset patients, there is a need for a holistic and multidimensional approach.

For example, in a recent systematic review of 14 psychosocial interventions for CRC patients,^31^ interventions found to be effective included written and verbal emotional expression, self‐efficacy enhancing interventions, Eastern Body‐Mind‐Spirit interventions, nurse‐administered information packets on rectal cancer and an intimacy enhancement intervention for patient‐partner dyads. However, on their own, these individual interventions only address a single psychosocial domain. There is also a lack of psychosocial interventions that addresses unique psychosocial concerns relating to CRC such as sexual dysfunction and embarrassing side effects due to treatment. Additionally, almost half of the interventions analysed did not have a significant effective on the psychosocial outcome examined. A holistic multidimensional approach can consider combining some of the effective interventions and incorporating ones targeted at the unique psychosocial concerns or unmet needs that young CRC patients experience.

Lastly, certain treatment‐related and socioeconomic factors can also affect psychosocial experience of young‐onset CRC patients. Consistent with previous studies on CRC treatment,^32^, ^33^ the need for additional treatment such as chemotherapy and stoma were also associated with poorer outcomes in young‐onset CRC patients. For young‐onset CRC patients, given that they are often diagnosed at a more advanced stage,^34^ the intensity of their treatment plans will consequently be greater and may therefore contribute to greater psychosocial impact. Lastly, it is worth noting the findings that patients with specific marginalised socioeconomic backgrounds, such as being female, having lower educational attainment, and living in deprived areas, were at risk of experiencing greater psychosocial impact. Identifying young CRC patients with these characteristics will be useful in providing additional support to particularly high‐risk groups.

### 4.1 Limitations of the included studies

Several crucial limitations were identified across the included studies in the current review. First, despite the fact that guideline for young‐onset CRC include those less than the age of 50 years at diagnosis,^35^ the upper age limit of the younger sample or sub‐sample across the included studies was inconsistent, ranging from less than 40 years up to 65 years. This heterogeneity of the age stratification in each study raises concerns on whether the results of studies with older upper age limits are truly representative of the young‐onset CRC experience.

Second, while discussion on the methodology used was limited given the small number of studies available, we noted the lack of quantitative scales specifically validated for CRC patients. Only one scale used the EORTC QLQ‐CR29^36^ which measures QOL domains characteristic to CRC. The inclusion of cancer‐specific scales is essential in uncovering psychosocial experiences unique to CRC, such as the influence of stoma, sexual dysfunction, and embarrassment with bowel movements. Another validated scale that future studies could consider is the Functional Assessment of Cancer Therapy‐Colorectal (FACT‐C) questionnaire,^37^ which was not used by any studies in this review.

Next, the majority of studies only measured the scores of participants at one timepoint, with participants being measured over a wide time period of less than 1 year to more than 5 years since diagnosis. This fails to account for possible changes in psychosocial experience from early in the diagnosis phase to after treatment, which has been shown to follow different trajectories depending on the characteristics of the CRC patients.^38^, ^39^, ^40^ This further highlights the lack of longitudinal designs in understanding the psychosocial experience of young‐onset CRC patients over time.

Lastly, six out of seven included studies were conducted in predominantly Western settings. Given that the diagnosis and treatment experience of CRC can be influenced by sociocultural contexts,^41^, ^42^, ^43^ the currently available literature is limited regarding the psychosocial experience of young‐onset CRC patients outside of the Western context. Future studies can consider looking into whether the psychosocial experience of young‐onset CRC in Asian or multi‐ethnic populations is similar to the findings in this review.

### 4.2 Limitations of the current review

Despite the broad search strategy utilised to capture an accurate state of the current literature available on the psychosocial experience of young‐onset CRC, inevitably some relevant texts may have been missed due to the fact only articles with full texts available in English are included. However, given that only four articles were excluded from the final sample due to full text not being available in English, the impact of this limitation is likely to be minor. Secondly, some articles may have been missed out as part of the screening procedure. There is a possibility that some articles on CRC patients may have reported relevant findings by stratifying results by age in the full text but did not mention it in the abstract. However, given that hand search of the reference lists of included studies was also conducted, the review has attempted to capture as many relevant articles as possible. While a broader search strategy was utilised, despite using the NCI definition for psychosocial experiences as a guide in the eligibility screening of records, perhaps more relevant articles might have been discovered if the search strategy outright included keywords derived from this NCI definition. As the body of literature on young‐onset CRC and psychosocial impact grows, future reviews should consider developing a more streamlined or specific search strategy. On the whole, despite the limitations, our review highlights the existing findings and gaps in literature which can guide the direction of future research.

Currently, there are no interventional studies on young CRCs targeting psychosocial issues associated with the treatment. While there exist interventional studies for young cancer in general on psychosocial domains such as fertility, body image and sexual health,^44^ they do not cover colorectal‐specific issues. For example, some interventions studies specified to CRC have looked into stoma‐related psychosocial outcomes, management and sexual dysfunction.^45^, ^46^ Hence, this review highlights the need to develop and test psychosocial interventions specific to young‐onset CRC.

## 5 CONCLUSION

In summary, this review represents a “first look” into the potentially differing psychosocial experience of suffering from CRC as a young‐onset patient. Collectively, the articles in our review suggest that young‐onset CRC patients may consistently face more severe impact on well‐established patient‐reported outcomes such as QOL, but also in challenges unique to this age group, such as self‐image and sexual functioning. With this in mind, clinical and allied health services should consider tailoring social support services and resources to recognise the unmet needs of young‐onset CRC patients. However, the current literature remains limited. From a research standpoint, a crucial gap that remains to be filled is to understand how these psychosocial experiences as well as other domains impact patient‐reported and clinical outcomes in younger patients over the course of treatment as well as survivorship.

## Funding information

This work was supported by the Singapore Ministry of Health's Clinician Scientist Award (Investigator Category) [MOH‐000333 (CSAINV19may‐0009)].

## CONFLICT OF INTEREST

The authors declare that there is no conflict of interest.

## Data Availability

Data sharing not applicable to this article as no datasets were generated or analysed during the current study.
